# The Heme Oxygenase/Biliverdin Reductase System and Its Genetic Variants in Physiology and Diseases

**DOI:** 10.3390/antiox14020187

**Published:** 2025-02-06

**Authors:** Cesare Mancuso

**Affiliations:** 1 Fondazione Policlinico Universitario A. Gemelli IRCCS, Largo F. Vito, 1, 00168 Rome, Italy; cesare.mancuso@unicatt.it; 2 Department of Healthcare Surveillance and Bioethics, Section of Pharmacology, Università Cattolica del Sacro Cuore, Largo F. Vito, 1, 00168 Rome, Italy

**Keywords:** cancer, cardiac diseases, gene regulation, personalized medicine, Parkinson’s disease, polymorphisms

## Abstract

Heme oxygenase (HO) metabolizes heme into ferrous iron, carbon monoxide (CO), and biliverdin-IXα (BV), the latter being reduced into bilirubin-IXα (BR) by the biliverdin reductase-A (BVR). Heme oxygenase exists as two isoforms, HO-1, inducible and involved in the cell stress response, and HO-2, constitutive and committed to the physiologic turnover of heme and in the intracellular oxygen sensing. Many studies have identified genetic variants of the *HO/BVR* system and suggested their connection in free radical-induced diseases. The most common genetic variants include (GT)n dinucleotide length polymorphisms and single nucleotide polymorphisms. Gain-of-function mutations in the *HO-1* and *HO-2* genes foster the ventilator response to hypoxia and reduce the risk of coronary heart disease and age-related macular degeneration but increase the risk of neonatal jaundice, sickle cell disease, and Parkinson’s disease. Conversely, loss-of-function mutations in the *HO-1* gene increase the risk of type 2 diabetes mellitus, chronic obstructive pulmonary disease, and some types of cancers. Regarding *BVR*, the reported loss-of-function mutations increase the risk of green jaundice. Unfortunately, the physiological role of the HO/BVR system does not allow for the hypothesis gene silencing/induction strategies, but knowledge of these mutations can certainly facilitate a medical approach that enables early diagnoses and tailored treatments.

## 1. Introduction

**Figure 1:**
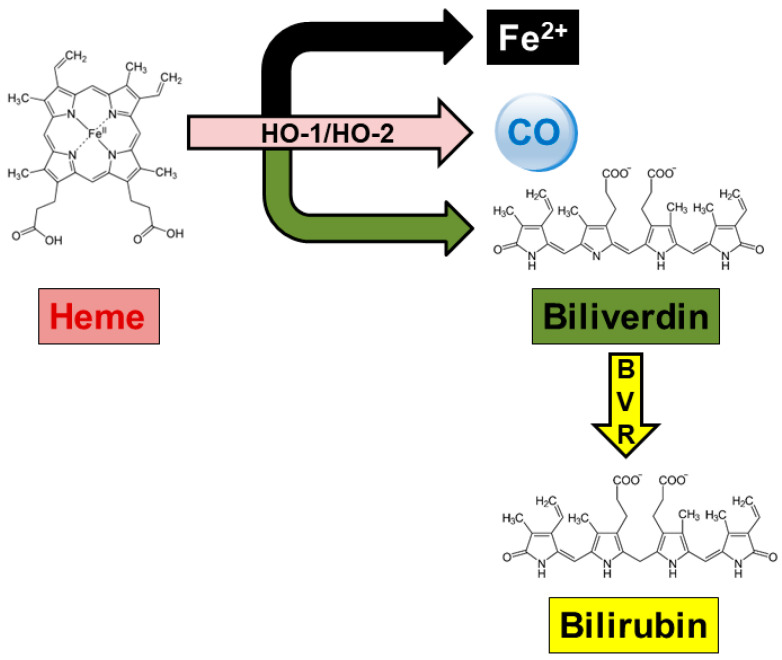
The heme oxygenase/biliverdin reductase system. Heme oxygenase (HO) isoforms, known as HO-1 and HO-2, catalyze heme oxidation into ferrous iron (Fe^2+^), carbon monoxide (CO), and biliverdin. The latter is then reduced by biliverdin reductase (BVR) into bilirubin, which is the final product of heme metabolism in mammals. For further information, see Section 2.1. Reproduced with permission from Mancuso, C. *Front Pharmacol* 2023 [2].

Heme oxygenase (HO) catalyzes the oxidation of heme moieties and the resulting formation of equimolar amounts of biliverdin-IXα (BV), carbon monoxide (CO), and ferrous iron (Fe^2+^) [1,2] (Figure 1). Heme oxygenase exists as two isoforms, HO-1 and HO-2, which catalyze the same reaction but are differently regulated and distributed [3,4]. Heme oxygenase-1 is inducible, almost ubiquitous, and involved in the cell stress response, whereas HO-2 is constitutive, greatly abundant in neurons, and involved in both the physiologic turnover of heme and intracellular oxygen sensing [2,3,4,5,6]. Downstream of HO is biliverdin reductase-A (BVR), a pleiotropic enzyme that has both reductase and tyrosine/serine/threonine kinase activities [7,8,9,10]. Through the reductase activity, BVR transforms BV into bilirubin (BR), a scavenger of reactive oxygen and nitrogen species (ROS and RNS, respectively), whereas due to the kinase activity, BVR is at the crossroad of many second messenger systems and cooperates with CO in regulating intracellular trafficking [5,11,12].

The interest of researchers in the HO/BVR system broke out in the early 1990s, following the discovery of the *HO-1* gene inducibility [3,13,14]. Based on this evidence, a wide array of redox-sensitive transcription factors has been identified. These factors translocate to the nucleus in response to inducers (e.g., physico-chemical stimuli, proinflammatory cytokines, and dietary polyphenols), bind specific sites in the *HO-1* promoter and enhance gene transcription [15,16,17]. Nevertheless, either excessive or prolonged HO-1 induction is harmful because it deprives cells of heme, a prosthetic group, and a cofactor for cytochromes and other hemoproteins and produces excessive CO and BR that become proinflammatory and neurotoxic [18,19,20,21]. With regard to constitutive HO-2, few studies have shown that this isoform is inducible by hypoxia, glucocorticoids, and morphine [22,23,24,25]. Indeed, Raju et al. described a glucocorticoid-responsive element (GRE) in the *HO-2* gene promoter of rat brain, thus supporting previous evidence showing the modulatory role of endogenous CO on the hypothamus−pituitary−adrenal axis [26,27,28]. Although BVR was initially considered a non-inducible protein, later studies showed that both proinflammatory molecules and xenobiotics induce this enzyme through transcriptional and non-transcriptional mechanisms [29,30,31].

The upregulation or repression of the HO/BVR system depends not only on the specific stressor, the transcriptional activator involved, and the duration of the stimulus but also on inherited factors. Researchers have identified two main variants in the 5′ flanking region of the *HO-1* gene, a (GT)n dinucleotide length polymorphism (*rs3074372*) and a T(−413)A single nucleotide polymorphism (SNP) (*rs2071746*) [32,33,34]. These highly polymorphic variants affect lung function and increase the risk of cancer or cardiovascular diseases in Asian ethnicity [33,35,36]. As far as the *HO-2* gene is concerned, two functional variants have been described in depth, namely the −42+1444A>G (*rs2270363*) and the *rs4786504*T>C [37,38]. These SNPs have been involved in the regulation of ventilator responses to hypoxia in Tibetan and Spanish populations and associated to the reduced risk of developing age-related macular degeneration (AMD) and increased risk of Parkinson’s disease (PD) in Caucasian individuals [37,38,39,40]. A third genetic variant of the *HO-2* gene has also been reported: the c.544G>A polymorphism (*rs1051308*), located in the c.544 position of the coding sequence inside the 3′-untranslated region (3′-UTR) [41,42]. Although the *HO-2* c.544G>A polymorphism may affect the stability of the transcript and the process of translation, its functional role is still controversial [41,42]. Ultimately, two variants in the *BVR* gene were defined: the Arg18X and c.214C>A SNPs. The Arg18X is characterized by a 52C-T transition in exon 2, resulting in an Arg18-to-ter (R18X) substitution predicted to truncate the protein N-terminal to the active site Tyr^97^, whereas the c.214C>A polymorphism consists of a 214C-A transversion in exon 3 of the *BVR* gene, resulting in a Ser^44^-to-ter (S44X) substitution [43,44]. Studies by Gåfvels et al. and Nytofte et al. demonstrated that these genetic variants increase the risk of hyperbiliverdinemia (green jaundice) in Swedish and Inuit individuals [43,44].

The goal of this paper is to provide a focused overview on the role of the previously mentioned highly polymorphic genetic variants in the expression of *HO-1*, *HO-2*, and *BVR* genes. In addition, the paper will also describe the contribution of these polymorphisms to the development of human diseases. Lastly, the review will analyze those classes of drugs whose therapeutic effect could be influenced by *HO-1*, *HO-2*, and *BVR* polymorphisms.

## 2. The HO/BVR System: Biochemical Characterization and Determinants of Function

### 2.1. Heme Oxygenases

As mentioned above, HO-1 and HO-2 catalyze the oxidative cleavage of the α-meso-carbon bridge of heme moieties in a four-step, oxygen-dependent process [3]. Reducing equivalents, provided by the NADPH-cytochrome-P-450 oxidoreductase (CYPOR), which transfers electrons from NADPH to heme, are necessary for oxygen activation and reduction of Fe^3+^ to Fe^2+^, as well as for maintenance of iron in the ferrous form [3]. Firstly, one electron reduces the heme−Fe^3+^−HO complex to heme−Fe^2+^−HO. In the following step, the HO activity, in the presence of both oxygen and a second electron, transforms heme−Fe^2+^ into the intermediate product α-meso-hydroxyheme. Thirdly, HO oxidizes α-meso-hydroxyheme into verdoheme, leading to CO release. Lastly, in the presence of oxygen and an additional electron, HO releases Fe^2+^ and transforms verdoheme into BV [45,46]. Importantly, several genetic variants of *CYPOR* may affect HO-1 enzymatic activity with mechanisms involving an altered binding of either flavin adenine dinucleotide or flavin mononucleotide (FMN) to the reductase (see Section 3.1.1). Heme oxygenase-1 is nearly ubiquitous, but it is particularly abundant in the spleen, cardiovascular system (including the atrioventricular node and myocytes, kidneys, and vessels, but only under redox imbalance), brain (hilus of the dentate gyrus, ventromedial and paraventricular hypothalamic nuclei, glial cells, and astrocytes), testes (Sertoli and Leydig cells), and gastrointestinal tract [3]. Heme oxygenase-2 has been detected in the brain (olfactory bulb, pyramidal neurons in the CA1-CA3 areas, cerebellum, and brainstem), testes (germ cell line, mature spermatocytes, and Leydig cells), and, to a lesser extent, in the liver, blood vessels (endothelial and smooth muscle layers), retina, and gastrointestinal tract [2,3].

The first evidence of a major role of endogenous CO was in the early Nineties, when several authors demonstrated its effects in the modulation of synaptic plasticity, nociception, neuropeptide release, non-adrenergic non-cholinergic relaxation, immune response, and renal function [27,47,48,49,50,51,52]. About 10 years later, the discovery of CO-releasing molecules, a class of compounds that release CO in aqueous solutions, contributed to further unraveling the innovative effects of CO in inflammation, apoptosis, myocardial damage, glucose metabolism, and cancer [53,54,55]. Carbon monoxide exerts these effects through the modulation of several intracellular systems and transcription factors (Table 1).

On the other hand, for many years, BV has been considered a waste product of HO activity. Subsequently, preclinical studies demonstrated how BV not only scavenges free radicals but modulates many signal transduction pathways, thus exerting beneficial roles in ischemia/reperfusion-related diseases, inflammatory diseases, graft-versus-host disease, viral infections, and cancers [76] (Table 2).

### 2.2. Biliverdin Reductase

Biliverdin reductase is a pleiotropic enzyme with both reductase and tyrosine/serine/threonine kinase activities. These two activities are strictly related, because the reduction of BV into BR can only occur, following the phosphorylation of BVR by itself or other kinases [76,88,89]. Through the reductase activity, BVR reduces the C10 methene (γ bridge) of BV, converting the linear tetrapyrrole into BR [90]. The reaction requires either NADH or NADPH at acidic pH or alkaline pH, respectively, and free thiols [89,91]. Biliverdin reductase is co-expressed with HO-1 or HO-2 in several organs, such as rat brain (neurons and glial cells) and kidneys, porcine and human gastric fundus (mucosal epithelial cells and endothelium of intramural vessels), and mouse liver and spleen (reticulo-endothelial/macrophage populations) [80,81,85,92,93].

BR exerts cytoprotective effects by scavenging free radicals, stimulating extracellular signal-regulated kinase (ERK)1/2 phosphorylation, enhancing the immune response and protecting cells exposed to neurotrophin deficiency [11] (Table 3). However, as mentioned above, excess BR is cytotoxic, in particular for the nervous system, where it increases glial apoptosis and impairs synaptic activity (Table 3).

These dual effects of BR have been confirmed in humans. At physiologic concentrations, by inhibiting oxidative stress and inflammation, BR protects against atherosclerosis, coronary heart disease, and peripheral artery disease [104,105,106]. Conversely, BR plasma levels greater than 300 µM (18 mg/100 mL) lead to kernicterus, a severe disease responsible for extrapyramidal abnormalities and sensorineural hearing loss in newborns [107].

**Figure 2:**
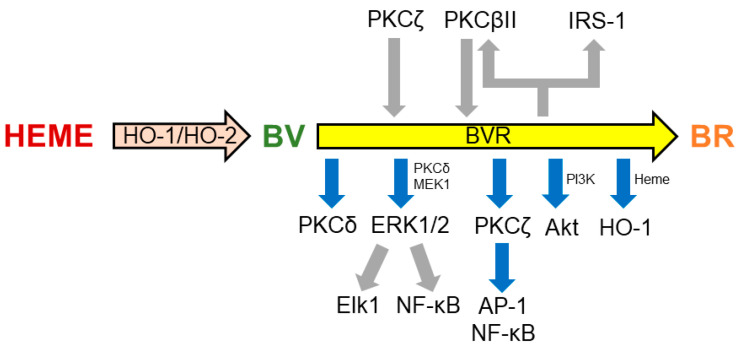
Some of the main targets of biliverdin reductase (BVR). A series of bidirectional phosphorylations and protein/protein interactions underlie the regulation of members of several kinase families by BVR. For further details, see Section 2.2. Gray arrows indicate phosphorylation; blue arrows indicate activation or protein/protein interaction. AP-1, activator protein-1; Elk1, ETS like-1 protein; ERK, extracellular signal-regulated kinase; IRK-1, insulin receptor kinase-1; IRS-1, insulin receptor substrate-1; MEK, mitogen-activated protein kinase kinase; PI3K, phosphatidylinositol 3-kinase; PKC, protein kinase C; PMA, phorbol myristate acetate; TNF-α, tumor necrosis factor-α.

Through the kinase activities, BVR regulates many cell-signaling pathways (Figure 2). After being phosphorylated by the insulin receptor kinase-1 on Tyr^198^, Tyr^228^, and Tyr^291^, BVR phosphorylates the human insulin receptor substrate-1 on Ser^307^, Ser^312^, and Ser^616^, thus blocking insulin action. These findings, along with the evidence that *BVR* silencing with siRNA is able to restore insulin-mediated glucose uptake, suggest a pivotal role of the reductase in glucose metabolism and insulin resistance [108] (Figure 2). By phosphorylating Thr^500^, BVR activates the typical protein kinase C (PKC) βII, which, in turn, may activate BVR under harsh conditions for autophosphorylation [8,109] (Figure 2). As far as the atypical PKCζ is concerned, this isozyme phosphorylates BVR at Ser^149^ and Ser^230^, but it is not a substrate for the BVR kinase activity. Evidence that BVR associates to PKCζ, in response to tumor necrosis factor-α (TNF-α), and enhances PKC activity towards its substrates suggests a novel role for BVR as an adaptor protein, linking PKCζ to proinflammatory transcription factors, such as the activator protein-1 (AP-1) and nuclear factor-κB (NF-κB) [8,110] (Figure 2). Once more, by a protein/protein interaction, BVR associates with PKCδ in response to insulin growth factor-1 and phorbol myristate acetate, resulting in an increased PKC activity [111]. Biliverdin reductase plays a role in the mitogen-activated protein kinase kinase (MEK)-ERK-Ets like protein (Elk) signaling pathway, promoting the formation of ternary complexes with either MEK1 and ERK1/2 or PKCδ and ERK2 [111,112]. The presence of BVR in these complexes is crucial because ERK1/2 is not able per se to move to the nucleus and, therefore, requires BVR, which instead has both nuclear localization and nuclear export motifs [112] (Figure 2). Once in the nucleus, ERK1/2 phosphorylates Elk1 and NF-κB and increases the transcription of antioxidant genes, including *HO-1* [8,111]. This shuttle activity also allows BVR to drive heme into the nucleus, providing a second mechanism for further HO-1 overexpression [113] (Figure 2). By interacting with PKC isoforms, BVR regulates inflammation and cell proliferation, and it is involved in breast cancer and tamoxifen resistance, PD, and type-2 diabetes mellitus (T2DM) [8]. Lastly, BVR activates the p85 subunit of phosphatidylinositol 3-kinase (PI3K), thus favoring the activation of PI3K and then Akt [8,80] (Figure 2). Through this pathway, BVR triggers pro-survival pathways downstream to Akt, enhancing cytoprotection, in particular in the nervous system.

## 3. The HO/BVR System: Gene Regulation and Genetic Variants

### 3.1. Heme Oxygenases

Heme oxygenase-1 and HO-2 are encoded by different genes located on chromosomes 22q12 and 16p13.3, respectively [114]. The human, mouse, and rat *HO-1* and *HO-2* genes are quite similar, 12–14 kb long and organized in five exons and four introns [6,17,115]. The HO-1 protein (molecular weight: 30–33 KDa) is the transcription product of a single message of about 1.8 kb, whereas the HO-2 protein (molecular weight: 36 KDa) is the product of two transcripts of about 1.3 kb and 1.7–1.9 kb [3]. When compared, HO-1 and HO-2 share an overall similarity of about 43%, but the active core of both isozymes is a preserved 24-amino-acid segment that forms the hydrophobic heme-binding pocket in the folded protein [3,116]. Within this segment, found in evolutionarily diverse species including humans, birds, and cattle, the consensus sequence LLVAHAYTR is considered the HO signature [3]. Furthermore, a conserved histidine residue (His^151^ in the rat) is essential for HO-2 activity, whereas His^132^, the homologue in HO-1, facilitates heme breakdown [3].

#### 3.1.1. Heme Oxygenase-1

The *HO-1* gene is overexpressed in response to physical factors (heat shock and UV irradiation), hypoxia, heavy metals (arsenite and cadmium), bacterial lipopolysaccharide, free radicals, proinflammatory cytokines [interleukin (IL)-1, TNF-α, and interferon-γ (IFN-γ)], dietary polyphenols (curcumin, ferulic acid, and resveratrol), and drugs (antiepileptics, antidepressants, and antipsychotics) [3,5]. These stressors induce *HO-1* gene expression by acting at the promoter region. Three upstream enhancer clusters, located 0.5 kb (also known as PP cluster), 4 kb (also known as E1 cluster), and 10 kb (also known as E2 cluster) from the transcription start site, have been identified in the 5′-flanking region of the *HO-1* gene [17]. In particular, the PP cluster binds heat shock factor (HSF), AP-1, and NF-κB, which are involved in the heat shock-, heavy metal-, and cytokine-related *HO-1* induction. The E1 cluster binds HSF, AP-1, and nuclear factor erythroid 2-related factor 2 (Nrf2), whereas the E2 cluster binds AP-1, Nrf2, and hypoxia inducible factor-1 (HIF-1). Both the E1 and E2 clusters mediate the heat shock-, hypoxia-, arsenite-, cytokine-, hemin-, drug-, and polyphenol-related *HO-1* gene overexpression [15,17]. Due to prolonged exposure, however, some of these stressors (e.g., hypoxia or IFN-γ) may repress rather than increase *HO-1* gene expression to reduce heme consumption and avoid excessive BR and CO production [20]. *Heme oxygenase-1* repression is regulated by the Bach proteins (Bach1 and Bach2) that compete with Nrf2 for heterodimerizing with Maf proteins and binding to the antioxidant responsive elements located in the E1 cluster [17,117]. This is an effective protective mechanism, because, under prolonged redox imbalance, Bach1 expression is induced in many human cells and tissues and efficiently counteracts Nrf2-mediated HO-1 gene induction [118,119,120]. Post-transcriptional modifications, such as exon skipping or polyadenylation of primary transcripts, may lead to the formation of shorter HO-1 isoforms that are differentially modulated by inducers [17]. *Heme oxygenase-1* gene expression is also regulated by BVR through several mechanisms, including either the heterodimerization of the reductase with members of the AP-1 family or Bach1, as well as binding metalloporphyrins [10]. Lastly, BVR allows induction of *HO-1* gene expression by reducing BV intracellular levels [10].

The ability of the cell to overexpress the *HO-1* gene may also depend on hereditary factors. Two main genetic variants occur in the *HO-1* promoter region, such as a (GT)n dinucleotide length polymorphism (*rs3074372*) and a T(−413)A SNP (*rs2071746*). Shigeki Shibahara and co-workers identified a highly polymorphic *HO-1* allele characterized by (GT) repeats ranging from 15 to 40 in the Japanese population [34]. The distribution of these (GT) repeats is trimodal, and the frequency distribution allows for the classification of these *HO-1* alleles into three subclasses: class S characterized by <25 GT repeats, class M characterized by 25 ≤ GT < 30, and class L characterized by ≥30 GT repeats [33,34]. From a functional viewpoint, the alleles with (GT) repeats of <25 upregulate the transcription of *HO-1* promoter, thus favoring gene overexpression, whereas alleles with (GT) repeats of ≥30 downregulate *HO-1* gene expression [33]. In this regard, it has been demonstrated that (GT) repeats favor a Z-DNA conformation that represses the transcription activity of DNA [121,122]. As far as the T(−413)A SNP is concerned, the *HO-1* (−413)A allele increases promoter activity and gene expression more than the (−413)T allele [32,123].

As mentioned above, several genetic variants of *CYPOR* may affect HO-1 enzymatic activity, the most representative being the missense mutations A115V (345C>T in exon 5), Y181D (541T>G in exon 5), P228L (683C>T in exon 6), M263V (787A>G in exon 7), A287P (859G>C in exon 8), R457H (1370G>A in exon 11), Y459H (1375T>C in exon 11), and V492E (1475T>A in exon 12) [124,125]. All of these polymorphisms reduce BV production, compared to the wild-type gene, to varying extents, but only the Y181D variant, which affects CYPOR-FMN binding, completely inhibits HO-1 activity [125].

#### 3.1.2. Heme Oxygenase-2

Early studies hypothesized the refractoriness of the *HO-2* gene to stimuli, obviously because of its constitutive nature. However, later studies by Mahin Maines’ group showed that, in the promoter region, the rat *HO-2* gene contains a consensus sequence of the GRE (between nucleotides −9723 and −9716) that makes it sensitive to adrenal glucocorticoids [26,116]. However, this GRE is not a strong transcriptional promoter; therefore, HO-2 overexpression may also depend on both an increased translation rate and protein stabilization [3,25,26,126]. Nevertheless, HO-2 overexpression was detected in both dorsal horn neurons of mice that exhibited morphine tolerance and hippocampal neurons of rats exposed to emotional stress [24,127]. The mechanisms leading to HO-2 overexpression in these experimental models need further elucidation, but the HPA activation is undoubtedly relevant in this context [128,129].

Similar to *HO-1*, the *HO-2* gene undergoes genetic variants, the most common being the −42+1444A>G (*rs2270363*) and the *rs4786504*T>C polymorphisms. The −42+1444A>G polymorphism is located in the 5′ flanking region of the *HO-2* gene, and the G allele has been associated to an increased *HO-2* transcription, whereas the A allele has been associated to a reduced gene expression [38,40,130]. With regard to the *rs4786504*T>C genetic variant, this SNP is located within an alleged SP1-binding site in intron 1 of the *HO-2* gene. Luciferase analysis has shown that the C allele, which creates two SP1-binding sites, increases *HO-2* expression at a higher level than the T allele that results in only one SP1-binding site [37]. Actually, a third genetic variant of the *HO-2* gene has also been reported, the c.544G>A polymorphisms (*rs1051308*), associated to an increased risk of multiple sclerosis (MS), PD, and AMD in Chinese and Caucasian individuals [41,42,131]. Nonetheless, the functional significance of this genetic variant is still controversial due to the weak association of this polymorphism with MS and the lack of statistical significance in AMD [41,42].

### 3.2. Biliverdin Reductase

Biliverdin reductase is evolutionary preserved, and genomic data suggest a universal distribution of this gene in mammals, fish, amphibian, reptiles, and birds [10,79]. The average sequence identity among mammalian species is greater than 80% [10]. Human BVR is encoded by a single gene located on chromosome 7q22, about 17 kb long and containing eight exons, with the initiation codon in exon 2 [10,132]. In the rat, the BVR protein, with a molecular weight of 34 KDa, is the product of a single transcript of about 1.6 kb [10]. In the human *BVR* gene, the promoter region (about 836 bp) is proximal to exon 1 and contains consensus regulatory elements for NF-κB (two binding sites, but only one functional), the aryl hydrocarbon receptor (AhR; five binding sites), HIF (four binding sites), and cAMP response element-binding protein (CREB; two binding sites) [133]. The presence of these elements implies a possible overexpression or repression of human *BVR* gene in response to different stimuli. In particular, hypoxia has been shown to upregulate the human *BVR* promoter and increase protein synthesis, whereas TNF-α, a proinflammatory cytokine involved in NF-κB activation, downregulates the promoter and reduces protein synthesis [133]. The upregulation of BVR detected in the rat kidney exposed to lipopolysaccharide or bromobenzene seems to be secondary to post-transcriptional activation of the enzyme rather than an increased promoter activation [134].

Regarding the inherited factors involved in *BVR* gene expression, the previously described Arg18X and c.214C>A genetic variants generate a mutated protein with no residual enzyme activity [43,44].

## 4. The HO/BVR System Polymorphisms in Human Diseases

### 4.1. Heme Oxygenase-1

#### 4.1.1. Chronic Obstructive Pulmonary Disease

Early evidence on the involvement of *HO-1* genetic variants in human diseases dates back to 2000, when Yamada et al. reported that (GT)n dinucleotide repeat length polymorphism is associated with susceptibility to chronic pulmonary emphysema (CPE) in Japanese smokers [33]. These Authors demonstrated that the presence of the L allele of the (GT)n locus, in both the homozygous and heterozygous genetic models (LL and L/M genotypes), was significantly higher in smokers with CPE than in those without CPE [33]. Conversely, the S allele seemed to serve as a negative risk factor [33]. The involvement of the *HO-1* polymorphism in lung function was also confirmed in the Caucasian ethnicity. Guénégou et al. genotyped 749 French subjects and confirmed that the presence of an L allele increases the susceptibility to developing airway obstruction, especially in heavy smokers [135]. Based on these earlier reports, additional studies analyzed the involvement of *HO-1* genetic variants in chronic obstructive pulmonary disease (COPD), but with inconsistent results [136,137,138,139]. Some years later, Zhou et al. meta-analyzed nine studies (1447 patients with COPD and 891 controls) reaching the conclusion that the *HO-1* L allele increases the susceptibility, particularly in the Asian population, but not the severity of the disease [140]. A possible explanation for these findings is that patients harboring the L allele have a reduced ability to overexpress HO-1, whose protective effects in lung diseases have been extensively demonstrated [140,141,142,143]. Indeed, a significant reduction in proinflammatory cytokines and chemokines has been detected in the lungs of mice genetically engineered to overexpress HO-1 and exposed to hypoxia [144]. Among the products of HO activity, CO has been shown to inhibit the expression and activities of matrix metalloproteinases in the human lung epithelial cell line A549, as well as the proliferation of cultured human airway smooth muscle cells, by inhibiting both ERK phosphorylation and cyclin D1 expression and NADPH oxidase activity [145,146]. Intriguingly, these authors have reported a cytoprotective effect of BR in inhibiting ERK phosphorylation in human bronchial smooth muscle cells [147].

#### 4.1.2. Hyperbilirubinemia

Heme oxygenase provides BV that is then reduced by BVR to BR. Therefore, it is clear that upregulation of HO isoforms leads to an increase in BR levels, provided there is enough BVR activity in the cell. The connection between increased HO activity and hyperbilirubinemia has also been clinically confirmed, since Sn-mesoporphyrin-IX, a well-known HO inhibitor, reduces BR plasma levels in babies affected by hemolytic and Crigler-Najjar type I diseases [148,149]. The role of *HO-1* genetic variants in neonatal hyperbilirubinemia has long been debated as some studies showed its close association with (GT)n repeat length polymorphism, whereas others strongly denied it [150,151,152,153,154,155]. This controversy has been resolved by Zhou et al. who carried-out a meta-analysis of seven eligible clinical trials involving 584 children with neonatal hyperbilirubinemia and 1655 controls [156]. In this study, the authors reported a significant association between neonatal hyperbilirubinemia and the *HO-1* S allele of the (GT)n locus in the recessive (genotypes SS versus LS + LL), dominant (genotypes SS + LS versus LL), and homozygous (genotypes SS versus LL) genetic models [156]. The explanation of the mechanism underlying this association is obvious. Individuals with at least one S allele have increased transcription of the *HO-1* gene, resulting in an overproduction of BV, which is then transformed into BR.

#### 4.1.3. Coronary Artery Disease and Restenosis

Several studies suggested a significant association of both the (GT)n dinucleotide repeat length and the T(−413)A polymorphisms in the *HO-1* gene with coronary heart disease (CHD) and restenosis (RS) in patients who underwent percutaneous coronary intervention (PCI) [157,158]. However, other clinical trials did not support this evidence [159,160]. In an attempt to solve this conundrum, Zhang et al. meta-analyzed 23 studies involving 12,130 patients with CHD or RS and 14,181 controls and confirmed that Asian subjects carrying the SS genotype of the *HO-1* (GT)n locus have a lower risk of developing CHD than those with the SL + LL and LL genotypes [36]. This cardioprotective effect went lost in Caucasian individuals. In the whole population, patients carrying the *HO-1* S allele have a reduced RS risk after PCI with respect to those with the L allele (S versus L or SS versus LL genotypes) [36]. Similar to patients with CHD, a subgroup analysis confirmed a reduced risk of RS in Asian subjects with the *HO-1* (GT)n SS genotype compared to those carrying other genotypes (S versus L, SS versus SL + LL, SS + SL versus LL, and SS versus LL) [36]. Regarding the T(−413)A *HO-1* gene polymorphism, a significant reduction in CHD risk was found in individuals with the AA genotype compared to those with other genotypes (AA versus AT + TT and AA versus TT) [36]. The fact that the genetic variants mentioned above increase the activity of *HO-1* promoter confirms that HO-1 overexpression is cardioprotective in patients with CHD. Many experimental models have extensively studied the effects of the HO-1/BVR system and their products in both the heart and vessels. Carbon monoxide counteracted neointimal hyperplasia in rodent models of vascular disease by inhibiting both vascular leukocyte activation and smooth muscle cell proliferation [161,162]. Furthermore, CO prevented platelet-derived growth factor-stimulated migration of rat vascular smooth muscle cells through the inhibition of NADPH oxidase-1 [163]. Finally, CO exerted antiplatelet effects by inhibiting mitochondrial respiration, at the cytochrome c oxidase level, as well as glycolysis due to cytosolic NAD^+^ depletion [164]. Lastly, BR also plays a pivotal role in the cardioprotective effects of HO-1 by scavenging ROS and RNS, inhibiting the inflammatory response as well as thrombus formation [165,166,167].

#### 4.1.4. Type 2 Diabetes Mellitus

The main role of oxidative stress, inflammation, and apoptosis in the pathogenesis of diabetes mellitus has prompted researchers to address the potential cytoprotective role of HO-1 in this metabolic disease [168,169]. In particular, HO-1 expression was increased in plasma, lymphocytes, and monocytes but decreased in muscle samples of individuals with T2DM and its sequelae [170,171,172]. Conflicting results were also stemmed when analyzing studies describing the possible association between *HO-1* polymorphisms and T2DM. Song et al. and Lee et al. reported that the L allele of the HO-1 (GT)n locus increases the risk of T2DM in 1103 Chinese and 536 Korean patients [173,174]. On the contrary, Arredondo et al. described that the *HO-1* S allele of the (GT)n locus is associated with an increased risk of T2DM in 99 Chilean subjects [175]. This controversy was solved in a meta-analysis of 5 studies (1751 T2DM patients and 2902 controls, including 190 and 235 Caucasians, respectively) by Bao et al. who confirmed that subjects harboring the *HO-1* L allele of the (GT)n locus, in particular with the LL genotype, have an increased risk of T2DM [176]. These results support the hypothesis that a reduction in *HO-1* gene expression is a risk factor for T2DM. Actually, in an animal model of diabetes mellitus, such as Sprague-Dawley rats treated with streptozotocin, HO-1 downregulation increased both superoxide anion formation in aortas and urinary 8-epi-isoprostane PGF_2α_, whereas the upregulation significantly counteracted oxidative damage, as demonstrated by the reduction in these local and systemic biomarkers [177]. Furthermore, it is worth mentioning that both CO and BV promote pancreatic β-cell regeneration, increase insulin secretion and protect β-cell against pro-oxidant and inflammatory damage as well as apoptosis [65,76].

#### 4.1.5. Cancer

The involvement of HO-1 in the pathogenesis of cancer is still matter of debate. Several studies have described a dual role of HO-1 in tumor progression as well as in chemoresistance [178,179,180,181,182]. By depleting heme, which is toxic under imbalanced redox conditions, such as during tumorigenesis, and providing BV/BR and CO, HO-1 increases the cell stress response and favors cytoprotection. On the other hand, the same products of HO activity can promote tumor progression by facilitating angiogenesis and metastasis [183,184,185]. With regard to *HO-1* genetic variants and their involvement in cancer, many clinical studies have addressed this topic, but with inconsistent results. The presence of the *HO-1* L allele of the (GT)n locus has been associated with the occurrence of malignant mesothelioma and lung carcinoma in Japanese subjects, whereas the S allele has been associated with either malignant melanoma in Austrian individuals or gastric cancer in Iranian patients and Japanese women [186,187,188,189,190]. Conversely, Andersen et al. reported no association of *HO-1* genetic variants with colorectal cancer in Danish people [191,192]. With the purpose to clarify the role of *HO-1* genetic variants in cancer, Wang et al. meta-analyzed 14 studies (2471 cancer patients and 2654 controls) and found that patients from East Asia carrying the *HO-1* L allele of the (GT)n locus (LL and LL + LS genotypes) had higher susceptibility of developing cancer, in particular digestive tract cancers, than those with the S allele (SS and SS + SL genotypes) [35]. In addition, an increased risk of developing squamous cell carcinoma was detected in the overall population with LL and LL + LS genotypes [35]. No significant association was found with the T(−413)A polymorphism and the overall cancer risk [35]. Evidence that the *HO-1* L allele of the (GT)n locus is involved in cancer suggests a cytoprotective role for HO-1 induction in tumorigenesis. In this regard, CO has been shown to activate p38 mitogen-activated protein kinase that inhibits proliferation and increases cell death in cancers [66,183,193]. Furthermore, CO increases oxidative metabolism in tumor cells, which, in turn, decreases nucleotide and aminoacid synthesis, arrests the cell cycle and enhances mitochondrial-dependent ROS generation and cell death [66,183].

#### 4.1.6. Miscellanea

Sickle cell disease (SCD) is a form of hemolytic anemia characterized by a point mutation in codon 6 of the gene encoding for the β-globin, resulting in the substitution of glutamic acid with valine (E6V) [194]. Mutated hemoglobin undergoes polymerization at low oxygen tension, causing a distortion of red blood cells (RBCs) that acquire a C (or sickling) shape [194]. Because of this rigid conformation, sickled RBCs are trapped in vessels, especially smaller ones, clogging them and causing complications such as acute chest syndrome (ACS), stroke, and anemia, the latter related to an increased hemolysis [194,195]. Because of the heme-degrading activity and CO production, HO-1 reduces vascular inflammation and oxidative stress-related damage in preclinical models of SCD [3,196]. The *HO-1* S allele of the (GT)n locus has been associated with the highest percentage of mutated hemoglobin in 942 children (from United States, Canada, France, and United Kingdom) affected by SCD [195]. Nonetheless, children with the SS genotype have a lower hospitalization rate for ACS [195].

Spanish subjects harboring the *HO-1* T allele of the T(−413)A locus with the TT genotype displayed a lower risk of developing restless leg syndrome and essential tremors than controls [197]. However, the latter have been considered weak, and further studies are necessary to deepen the role of *HO-1 rs2071746* polymorphism and these neurological diseases.

As far as the *CYPOR* genetic variants mentioned above, they play a key role in the pathogenesis of Antley and Bixler syndrome, a rare pediatric condition whose main outcomes are skeletal malformations, abnormal steroid levels, and sexual ambiguity. In particular, the *CYPOR* Y181D mutation, which significantly compromises HO-1 activity, is characterized by a clinical phenotype with midface hypoplasia and phalangeal malformations [124]. Whether or not HO-1 downregulation plays a direct role in Antley and Bixler syndrome is still unclear, and it could open a new avenue in an unexplored field of research.

### 4.2. Heme Oxygenase-2

#### 4.2.1. Parkinson’s Disease

As previously described, the constitutive isoform is quite abundant in neurons, and the substantia nigra (SN), which contains the cell bodies of dopaminergic neurons forming the nigrostriatal tract, is among the brain areas with the highest HO-2 activity [3]. Ayuso et al., who genotyped 691 Spanish patients affected by PD for about 10 years and 747 control subjects, found a significant association of the *HO-2* G allele of the −42+1444A>G locus with these patients [38]. The GG genotype was significantly higher in PD subjects than in controls [38]. As mentioned above, the G allele in this polymorphism increases *HO-2* gene expression, leading to an upregulation of heme catabolism [38]. Evidence that PD subjects have an increased HO-2 expression supports the hypothesis that HO activity products may promote neurodegeneration [198]. Increased iron deposition, due to HO activity, was detected in both the SN and putamen of 32 patients affected by PD for more than 5 years [199]. Furthermore, increased BR plasma levels were found in 420 subjects with moderate PD for ~4 years [200,201]. It is not easy to address whether increased brain iron and plasma BR, detected in PD patients, play a causal neurodegenerative role or are linked to increased HO-1 expression in response to prooxidant conditions. If the neuroprotective role of CO described in many preclinical models of PD is added, the picture becomes even more complicated [202]. In any case, the evidence that a prolonged expression of HO-1 and accumulation of its products over time lead to neurotoxicity is beyond dispute. Therefore, it remains plausible that the increased expression of HO-2 detected in patients with PD for 4–5 years may have generated excess iron, CO, and BR responsible for neurodegeneration.

#### 4.2.2. Age-Related Macular Degeneration

Age-related macular degeneration is an eye disease characterized by a progressive loss of the central vision and the leading cause of visual impairment in Western countries. Oxidative stress and dysregulation of the immune-inflammatory response, mainly the complement system, play an important role in AMD pathogenesis [203,204]. Two forms of AMD have been described, the dry and the wet, the latter being the most severe form and involved in vision loss and blindness [205]. The GG genotype of the −42+1444A>G polymorphism of the *HO-2* gene has been associated with a reduced risk of developing dry AMD, although this genotype favors the progression of AMD from the dry to the wet form [40].

#### 4.2.3. Ventilator Response to Hypoxia

As reported earlier, together with the physiologic heme-metabolizing activity, HO-2 has an additional function, namely sensing intracellular oxygen levels [2,3,6,206]. This function was hypothesized because the 3′ UTR of HO-2 mRNA contains a sequence that completely overlaps with the oxygen-sensing consensus sequence 5′TTTTGCA3′ and HO-2 contains two additional heme-binding sites in the folded protein, different from the heme catalytic site and named heme regulatory motifs (HRMs) [3,207,208]. These HRMs, whose affinity for heme is much higher than that of the catalytic pocket, are common to other hemoproteins, such as catalase and ALA synthase, and confer a regulatory function to protein [209]. These early observations were confirmed in the following years by other studies showing that HO-2 is able to physically interact and regulate the large-conductance Ca^2+^-activated K^+^ (BKCa) channel in the carotid body [6,210,211]. Carotid bodies are important chemosensors and, by increasing both the rate and depth of ventilation, contribute to the systemic response to hypoxia [212].

Yang and colleagues have described the genetic mutation *rs4786504T>C* capable of altering HO-2 expression (see Section 3.1.2) in 1250 Tibetans, residing at altitudes between 4200 m and 5100 m [37]. A reduction of about 2 g/dL in circulating hemoglobin level was measured in male Tibetans homozygous for the C allele of *rs4786504* SNP compared with in the homozygous carriers of the T allele [37]. This decreased hemoglobin count is not surprising, because the C allele of *rs4786504* results in HO-2 upregulation and degradation of heme moieties, thus providing an endogenous negative feedback mechanism against polycythemia [37]. Although a transient raise in hemoglobin count is an acclimatization response to the reduced oxygen partial pressure, due to decreased barometric pressure, in travelers who climb high altitudes and in populations living permanently at high altitudes, hyperhemoglobinemia is an important risk factor for cardiovascular diseases. This is because it increases blood viscosity and impairs tissue blood flow and oxygen delivery [37,39,213,214]. An altered response to hypoxia, due to the *rs4786504* mutation in the *HO-2* gene, was also found in 84 Europeans residing at sea level [39]. Specifically, individuals homozygous for the C allele of the *rs4786504* SNP showed higher ventilatory responses to hypoxia than the T allele carriers (CT + TT) both at rest and during submaximal exercise [39]. However, no change in hemoglobin count was detected in this population. This is a relevant difference between European sea-level residents and Tibetans exposed to hypoxic conditions and should be explained on the basis of an increased red blood count due to chronic erythropoietin exposure in the Asian population.

Among the products of HO-2 activity, CO is involved in the oxygen-sensing function of this isoform. Under normoxic conditions, HO-2 binds heme with high affinity and generates CO, which, in turn, activates the BKCa channel that allows potassium to flow outward and resulting in cell membrane repolarization. On the contrary, reduced oxygen levels, such as during hypoxia, decreases the HO-2 affinity for heme, which results in reduced CO production and BKCa channel closure. This last event increases intracellular potassium concentration, leading to membrane depolarization. This hypoxic response enhances the release of acetylcholine, dopamine, and adenosine triphosphate in the carotid bodies and ultimately increases the ventilation rate and depth [6,210,215].

### 4.3. Biliverdin Reductase

#### Green Jaundice

Due to the co-localization of HO isoforms with BVR, BV does not accumulate in cells under physiological conditions, because it is reduced into BR by the BVR activity. Nevertheless, if BVR undergoes downregulation, hyperbiliverdinemia may occur.

A heterozygous nonsense mutation in exon 3 (Arg18X) of the *BVR* gene was described in a male Swedish patient with decompensated liver cirrhosis [43]. Interestingly, this patient developed severe hyperbiliverdinemia (green jaundice) in the presence of a functional allele, implying the main contribution of liver cirrhosis to the clinical syndrome [43]. This early observation was supported a few years later by Nytofte et al. who described the homozygous nonsense mutation c.214C>A (p.Ser44X) in the BVR gene, which results in a non-functional enzyme, in two unrelated Inuit women with biliary obstruction [44]. Both of these women revealed hyperbiliverdinemia and hyperbiliverdinuria [44]. Five years later, the same research group demonstrated that the c.214C>A mutation is common in Inuits living in Greenland (1% homozygous and 4.5% heterozygous, with a total allelic frequency for the mutation of 5.47%) [216]. However, patients carrying the *BVR* genetic variants reported above would not show an abnormal phenotype because BV itself may undergo biliary excretion provided that the biliary function is preserved [44].

## 5. Potential Impact of Genetic Variants in the HO/BVR System on the Effect of Drugs

By binding the AhR, BV upregulates the 1A1/1A2 isoforms of cytochrome P-450 (CYP1A1/1A2) [82]. On these grounds, BV behaves as an endogenous enzymatic inducer, a substance that decreases the pharmacologic effects of drugs whose metabolism depends on the CYP1A1/1A2 isoform. For this reason, those individuals harboring either the *HO-1* S allele of the (GT)n locus or the A allele in the T(−413)A locus, both polymorphisms leading to an increased BV production due to HO-1 overexpression, may have reduced pharmacological effects of several drugs metabolized by the CYP1A1/1A2 isoform, including acetaminophen, theophylline, warfarin, clozapine, and others.

Since HO-1 overexpression promotes chemoresistance of cancer cells, tumors in which the *HO-1* L allele of the (GT)n locus has been detected (e.g., malignant mesothelioma, lung carcinoma, and digestive tract cancers) may have a better therapeutic response towards some antiblastic drugs, such as daunorubicin, gemcitabine, and cisplatin [183,217,218,219].

## 6. Conclusions and Future Directions

When the first evidence appeared regarding the impact of *HO-1* genetic variants on the pathogenesis of lung diseases, the scientific community became interested in the potential role of these polymorphisms as disease biomarkers. The possibility of identifying the risk of undergoing neurological, cardiological, or metabolic diseases from a simple blood sample, even taken during childhood, was a mesmerizing prospect. In addition, the prevalence of certain polymorphisms in particular ethnic groups favored the implementation of basic concepts of precision medicine, such as early diagnosis and tailored therapy, in free radical-induced diseases. Unfortunately, these hopes did not lead to the expected results. Molecular biology techniques necessary for genotyping, including the most recent next-generation sequencing technology, are not always available in many developing countries and are often unaffordable by a large portion of the population. These limitations have been a major barrier to further investigation of the role of *HO/BVR* polymorphisms as disease predictors. In addition, the conflicting results about the association of *HO-1* genetic variants with COPD, neonatal jaundice, T2DM, and cancer, as well as the reduced sample size of clinical studies that have associated *HO-2* polymorphisms with PD and AMD, do not enable drawing conclusions on the impact of *HO* genetic variants and these diseases.

Concerning the impact of HO/BVR modulation on the effect of drugs, the dual nature of the products of their enzymatic activities yields limited information. As reported in previous sections, Fe^2+^, BV, CO, and BR have physiological and sometimes protective effects on many tissues and cell types, but they can become toxic if produced in excess. Therefore, the ideal approach would be to identify agents that increase the HO and BVR expression without exceeding the toxicity threshold of their by-products and for a controlled period. This goal is nearly impossible to achieve because the previously assumed threshold and period are not predetermined and may vary from tissue to tissue and cell to cell. In addition, the availability of phototherapy in neonatal jaundice and several effective drug classes for the prevention and treatment of COPD, T2DM, and RS following PCI do not allow for the consideration of HO-1 modulation as an unmet medical need.

In conclusion, clinical studies showing the role of the genetic variants of *HO-1*, *HO-2*, and *BVR* have helped shed new light on the involvement of these genes in many disorders, but their impact as disease modifiers has not yet been defined and will require more effort by basic and clinical researchers. In particular, the availability of suitable animal models ensures that the potential beneficial role of *HO/BVR* gene modulation, and its possible translational impact in free radical-induced diseases, can be early investigated at the preclinical stage. In addition, knowledge of these mutations and associated risks can certainly facilitate a medical approach that allows early diagnosis and tailored treatments.

## Figures and Tables

**Table 1:** Some of the main carbon monoxide (CO) targets.

Target	Effect(s)	Main Outcome(s)	Reference(s)
Soluble guanylyl cyclase	Stimulation of cGMP production	Modulation of synaptic plasticity and olfactory function; NANC relaxation; inhibition of VSMC proliferation; inhibition of platelet aggregation; stimulation of insulin release	[51,52,56,57,58,59]
Cyclooxygenase PGE_2_	Stimulation	Inflammation; neuropeptide release	[60,61]
BKCa channel	Opening/activation	Vasodilation; regulation of ventilator responses	[62,63,64]
Kir2.3 channels	Inhibition	Increased insulin secretion	[65]
Mitochondria	Increased ROS generation Increased ATP production	Cancer cell death Increased insulin secretion	[65,66]
TLR4	Overexpression	Chemoresistance	[67]
NF-κB	Activation	Inhibition of endothelial apoptosis	[68]
MAPK p38	Activation	Inhibition of endothelial apoptosis; stimulation of apoptosis in cancer cells	[66,69]
PDGF-B	Inhibition	Inhibition of inhibition of VSMC proliferation	[70]
VEGF	Activation	Vasculogenesis and myocardial regeneration	[71,72]
NADPH oxidase	Inhibition of ROS production	Alleviation of vascular inflammation; inhibition of VSMC proliferation; improvement of brain endothelial cell survival	[53,73,74]
Soluble β-amyloid	Reduction of oligomerization	Slowing of neurodegeneration	[75]

ATP, adenosine triphosphate; BKCa channel, large-conductance Ca^2+^-activated K^+^ channel; Kir2.3 channels, inward rectifying K^+^ channels 2.3; MAPK, mitogen-activated protein kinase; NANC, non-adrenergic non-cholinergic; NF-κB, nuclear factor-κB; PDGF, platelet-derived growth factor; PG, prostaglandin; ROS, reactive oxygen species; TLR, toll-like receptor; VEGF, vascular endothelial growth factor; VSMC, vascular smooth muscle cell.

**Table 2:** Some of the main biliverdin (BV) targets.

	Effect	Main Outcome(s)	Reference(s)
ROS/RNS	Scavenging	Antioxidant effect	[77,78]
BVR	Activation	BR production and free radical scavenging	[78]
PKCδ	Inhibition	Downregulation of proinflammatory NF-κB, IL-6, TNF-α and iNOS	[8,79]
PI3K/Akt	Activation	Release of antinflammatory IL-10	[80]
eNOS	Activation	NO release and S-nitrosylation of BVR that inhibits TLR4 expression	[81]
AhR	Activation	CYP1A1/1A2 overexpression; amplification of TCDD toxicity	[82,83]
Soluble guanylyl cyclase	Stimulation	Potentiation of CO-dependent relaxation in pig gastric fundus	[84,85]
VEGF-A	Overexpression	Acceleration of angiogenesis in CRC.	[86]
EGFR	Downregulation	Inhibition of tumor growth in head and neck carcinoma	[87]

AhR, aryl hydrocarbon receptor; BR, bilirubin; BVR, biliverdin reductase; CO, carbon monoxide; CRC, colorectal cancer; CYP, cytochrome P-450; EGFR, epidermal growth factor receptor; eNOS, endothelial nitric oxide synthase; IL, interleukin; iNOS, inducible nitric oxide synthase; NF-κB, nuclear factor-κB; NO, nitric oxide; PKC, protein kinase C; PI3K, phosphatidylinositol-3 kinase; RNS, reactive nitrogen species; ROS, reactive oxygen species; TCDD, 2,3,7,8-tetrachlordibenzo-p-dioxin; TLR4, toll-like receptor 4; TNF-α, tumor necrosis factor-α; VEGF, vascular endothelial growth factor.

**Table 3:** Some of the main bilirubin (BR) targets.

Target	Effect(s)	Main Outcome(s)	Reference(s)
Peroxyl radical Nitric oxide Nitroxyl anion Peroxynitrite	Scavenging	Antioxidant effect	[78,94,95]
MAPK ERK1/2 p38 JNK1/2	Activation	Neuroprotection through the nNOS/NO axis in PC12 and in primary cultures of CGN; neurotoxicity due to increased release of IL-1β, IL-6, and TNF-α in glial cells	[96,97,98]
NF-κB	Inhibition of nuclear translocation	Immunomodulation and protection from autoimmune disease.	[99]
Caspase-3	Activation	Apoptosis in PC12, neuronal and glial cell lines	[96,100]
NMDAR	Decrease in NR1, NR2A, and NR2B subunits	LTP/LTD impairment	[101]
NGF, BDNF	Inhibition of signaling pathways downstream (Akt/PKB)	Neurotoxicity in PC12 and in primary cultures of CGN	[96]
Mitochondria	Disruption of membrane	Increase in intracellular Ca^2+^ and cell death in glial cells	[102,103]

BDNF, brain-derived neurotrophic factor; CGN, cerebellar granule cells; ERK, extracellular signal-regulated kinase; IL, interleukin; JNK, c-Jun N-terminal kinases; LTD, long-term depression; LTP, long-term potentiation; MAPK, mitogen activated protein kinase; NF-κB, nuclear factor-κB; NGF, nerve growth factor; NMDAR, N-methyl-D-aspartate receptor; nNOS, neuronal nitric oxide synthase; NO, nitric oxide; PKB, protein kinase B; TNF-α, tumor necrosis-α.
